# Assessment of the Effect of High or Low Protein Diet on the Human Urine Metabolome as Measured by NMR

**DOI:** 10.3390/nu4020112

**Published:** 2012-02-20

**Authors:** Lone G. Rasmussen, Hanne Winning, Francesco Savorani, Henrik Toft, Thomas M. Larsen, Lars O. Dragsted, Arne Astrup, Søren B. Engelsen

**Affiliations:** 1 Department of Human Nutrition, Faculty of Life Sciences, University of Copenhagen, DK-1958 Frederiksberg, Denmark; Email: logr@life.ku.dk (L.G.R.); tml@life.ku.dk (T.M.L.); ldra@life.ku.dk (L.O.D.); ast@life.ku.dk (A.A.); 2 Foss Analytical A/S, DK-3400 Hillerød, Denmark; Email: hwi@foss.dk (H.W.); ht@foss.dk (H.T.); 3 Quality and Technology, Department of Food Science, Faculty of Life Sciences, University of Copenhagen, DK-1958 Frederiksberg, Denmark; Email: frsa@life.ku.dk

**Keywords:** metabolomics, metabolite profiles, dietary intervention, dietary protein, NMR spectroscopy, chemometrics

## Abstract

The objective of this study was to identify urinary metabolite profiles that discriminate between high and low intake of dietary protein during a dietary intervention. Seventy-seven overweight, non-diabetic subjects followed an 8-week low-calorie diet (LCD) and were then randomly assigned to a high (HP) or low (LP) protein diet for 6 months. Twenty-four hours urine samples were collected at baseline (prior to the 8-week LCD) and after dietary intervention; at months 1, 3 and 6, respectively. Metabolite profiling was performed by ^1^H NMR and chemometrics. Using partial least squares regression (PLS), it was possible to develop excellent prediction models for urinary nitrogen (root mean square error of cross validation (RMSECV) = 1.63 mmol/L; *r* = 0.89) and urinary creatinine (RMSECV = 0.66 mmol/L; *r* = 0.98). The obtained high correlations firmly establish the validity of the metabolomic approach since urinary nitrogen is a well established biomarker for daily protein consumption. The models showed that trimethylamine-*N*-oxide (TMAO) is correlated to urinary nitrogen. Furthermore, urinary creatine was found to be increased by the HP diet whereas citric acid was increased by the LP diet. Despite large variations in individual dietary intake, differentiated metabolite profiles were observed at the dietary group-level.

## 1. Introduction

Dietary assessment plays a crucial role in our ability to clarify relationships between dietary exposure and disease causation. Therefore, high-quality dietary information is a key issue in establishing causality in nutritional studies. The best approach to ascertain valid information of the dietary intake is within the context of prospective dietary intervention studies carried out under highly controlled conditions. However, well-controlled feeding studies are costly and therefore often contain a relatively small number of participants and have short duration. Thus, a considerable proportion of our knowledge relating dietary intake to phenotypes and disease risks comes from population studies using, for the most part, quantitative food diaries, dietary recalls or food frequency questionnaires. Despite being a well accepted methodology, these methods are often inaccurate as they are based on self-reported data and can be prone to misreporting because of inadequate recalls [1,2] and especially serious underreporting of energy intake has been observed in obese people [3,4].

A goal for nutritional metabolomics is to determine a quantitative link between intake of a particular nutrient or eating pattern to a particular metabolic profile. The application of metabolomic approaches may also help resolving some of the inherent inaccuracy in documenting dietary intake. Biomarkers of dietary protein and especially meat and vegetable specific biomarkers are of particular importance as a higher meat intake has been associated with several diseases including hypertension, type-2 diabetes, cardiovascular disease and cancer [5,6,7]. In a study by Stella *et al*. [8] they conducted a nuclear magnetic resonance (NMR) metabolomics investigation for the characterization of the metabolic effects of vegetarian, low meat, and high meat diets in humans. They found that the high meat content diet was associated with elevated urinary levels of creatinine, creatine, carnitine, trimethylamine-*N*-oxide (TMAO), taurine, and 1- and 3-methylhistidine when providing the 3 diets to twelve healthy men in a cross-over design. A study by Xu *et al*. [9] investigated the variability in the metabolic urinary profiles from subjects from either lacto-vegetarian or lacto-ovo-vegetarian diets and found that plant-based diets were characterized by increased urinary *N*-acetyl glycosamine (NAG), succinate, citrate, phenylalanine, formate and glycine. Nevertheless, it remains unclear how a specific dietary composition influences the metabolism and to what extent the turnover of low-molecular-weight metabolites are influenced in the long term. A high degree of compliance to the diet is necessary in order to obtain valid results, which requires study designs with strict control of the diet, and advanced technologies for metabolite analyses. Metabolomic studies on 24 h urine samples in long-term dietary intervention trials are still very scarce and much further research is needed. The aim of the present study was to identify the metabolites responsible for the discrimination between high vs. low intake of dietary protein in the diet after 1, 3 and 6 months, respectively, in a controlled dietary intervention when applying a holistic metabolomics approach where no *a priori* selection of metabolites was made.

## 2. Experimental Section

### 2.1. Study Design

The present study is an extension of the work that was carried out in the Pan-European DiOGenes study. The main aspects of the research conducted in the comprehensive, long-term, randomized, controlled dietary intervention study was to address the impact of dietary protein and glycemic index (GI) on weight (re)gain in a large number of families in which parents and children suffer from obesity or are overweight. A detailed description of the study and further information on the study recruitment, exclusion criteria and the investigations carried out at the clinical investigation days have been described in two methodological papers [10,11]. The study population in the present study consisted of the adult members of the families participating in the intervention study carried out in the Danish centre. The study is registered at ClinicalTrials.gov, number NCT00390637.

### 2.2. Subjects

**Figure 1:**
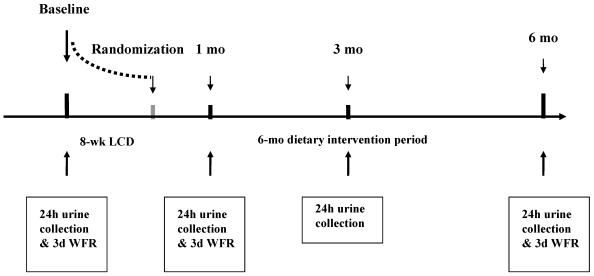
Timeline of the part of the DiOGenes study included in the present study and the scheduled 24 h urine collections and 3-day weighed food records (WFR). LCD, low-calorie diet; HP, high-protein; LP diet, low-protein diet.

A total of 109 healthy, obese with a body mass index 30.7–37.2 kg/m^2^, non-diabetic subjects aged 37–45 years underwent the clinical investigation day at baseline, which included collection of 24 h urine samples. All subjects then underwent an 8-week low-calorie diet treatment before starting the dietary interventions. Out of these 101 subjects collected 24 h urine samples after 1 month and 95 subjects collected 24 h urine samples after 3 months of the dietary intervention period. Finally 77 (44 women; 33 men) of these subjects also collected 24 h urine samples and underwent the clinical investigation day at month 6, at the end of the dietary intervention period. The metabolomic analyses in this study included only the 77 subjects who completed the 24 h urine collections at all four time points. A schematic presentation of the study design is given in Figure 1.

### 2.3. Experimental Design

The current study was designed as a parallel intervention trial with 5 dietary intervention groups and the subjects were randomly assigned to a 6-month low-fat (25–30% of energy) diet based on one of the following interventions: low protein, low GI (LP/LGI); low protein, high GI (LP/HGI); high-protein, low GI (HP/LGI) and high-protein, high GI (HP/HGI) or to a control (CTR) diet according to the current Danish official guidelines with an intermediate content of protein. For the demographic subject characteristics and the mean dietary macronutrient composition during the 6-month diet intervention as well as for the analysis aimed at biomarker identification, the two HP groups were pooled, as well as the two LP groups in order to average out the variation in carbohydrate intake. In this experimental design, 77 subjects were included (42 in the HP and 35 in the LP diet group). Furthermore, in these statistical and metabolomics analyses the control group (which included a total of 18 subjects) was left out due to the intermediate content of protein but the baseline measurements for each group were retained.

The study subjects were asked to complete a 3-day weighed food record (3d WFR) for three consecutive days, including two week days and one weekend day, both at baseline (prior to the 8-week LCD), and after randomization to the dietary intervention; at months 1 and 6, respectively. 

The subjects were instructed to weigh all their foods whenever possible and to supply information on brand names, cooking and processing. When weighing was not possible (e.g., when dining out) they were instructed to record the food in household measures (cups, glasses, tablespoons, *etc*.).

To mimic free-living conditions, the 6-month dietary intervention was based on an *ad libitum* design where the families were provided with most food, free of charge, from a trial supermarket at the department. The validated supermarket model has been described elsewhere [12].

A computer program (DiOGenes, version 1.4; Scientific Nutrition Supervision, Greve, Denmark) was constructed for recording of foods (product database) and for the calculation of nutrient composition of each shopping session during the 6-month dietary intervention. Local food manufacturers donated most products. Additional products were purchased to ensure an appropriate assortment to cover the dietary needs and the variability required by all diet groups throughout the 6-month period. The products consisted of a broad variety of all kind of foods, and thus the families were exposed to many different sources of protein.

The low protein diets were designed to be low in protein (10–15% of energy), whereas the high protein diets prescribed 23–28% of energy from protein. Regarding carbohydrate, the HP diets were designed to contain 45–50% of energy from carbohydrates, and the LP diets contained 57–62% of energy from carbohydrates. A detailed description of the diets and the dietary strategy is described elsewhere [11]. Alcohol consumption was allowed in accordance to the current guidelines issued by the Danish National Board of Health, *i.e.*, <14 units/week and <21 units/week (1 unit = 12 g alcohol) for women and men, respectively. Subjects were instructed to engage in minimum 30 min/day of moderate physical activity to achieve energy balance and weight maintenance, but the actual physical activity level was not reported by the subjects. All subjects were allowed a 3-week break from the project, during which no recording of the dietary intake was required. The 3-week break was randomly distributed throughout the 6 months among the study participants. However, the break was not allowed to be less than one week away from the 24 h urine collections and the corresponding 3-day dietary records.

### 2.4. 24-h Urine Collection and Storage

Twenty-four hour urine samples were collected both at baseline (prior to the 8-week LCD) and after randomization to the dietary intervention; at months 1, 3 and 6, respectively (Figure 1). The subjects were each provided with 2 sterile, pre-weighed and airtight 2.5 L polyethylene containers and a sterile 500 mL container. The subjects were instructed not to collect the first urine-void in the morning on the first day, but after this first void, the urine collection started and continued until and including the morning urine-void the following day. During the 24 h the subjects were instructed to store the containers in the fridge or in another cool place, whenever possible [13]. The subjects were also instructed to take a para-aminobenzoic acid (PABA) tablet (240 mg/day) at the morning meal, at lunch and at dinner, respectively, as a control of the completeness of urine collection, since PABA is absorbed and excreted in the urine within 24 h [14]. 

### 2.5. Urine Sample Handling

The total volume and density of the collected 24 h urine was determined and two 5 mL aliquots from each of the collections were drawn and stored at −80 °C until further preparation and analysis. Urinary creatinine concentrations were determined by a colorimetric assay (Ortho-Clinical Diagnostics, Johnson & Johnson, Birkeroed, Denmark) for the Vitros 5.1 FS analyzer. Urinary nitrogen was determined by Dumas combustion methodology, using a VarioMax CN analyzer (Elemtar, Hanau, Germany), and urinary PABA by spectrophotometry (Stasar, Gilford Instruments Laboratories, Oberlin, USA).

Prior to the NMR analyses of the urine, the samples were thawed at 4 °C, centrifuged at 4 °C at 1600 rpm for 10 min and then an 340 μL aliquot of the supernatant was added 170 μL of 100 mM phosphate buffer (pH 7.4) to reduce the pH range of the samples. Perdeuterated 3-(trimethylsilyl) propionate sodium salt (TSP) was added to act as an internal chemical shift reference (δ 1H 0.00), 10% (v/v) D_2_O was added to provide a lock signal for the NMR spectrometer and a 0.1% (w/v) NaN_3 _was added as a preservative. A Gilson cooling rack kept the samples cooled at 4 °C prior to injection. 

### 2.6. ^1^H NMR Analyses

^1^H NMR spectra were acquired on a Bruker DRX 600 MHz spectrometer (Bruker Biospin Gmbh, Rheinstetten, Germany) operating at 60,000 MHz for protons (14.09 Tesla) using a broadband inverse detection probe head equipped with a 120 μL flow-cell. Data were accumulated at 300 K employing a pulse sequence composed by a presaturation of the water resonance during the recycle period followed by a composite 90 degree pulse with an acquisition time of 2.73 s, a recycle delay of 2 s, 128 scans and a sweep width of 12019.23 Hz, resulting in 64 k complex data points. All samples were individually and automatically tuned, matched and shimmed. Prior to Fourier transformation, each free induction decay (FID) was zero-filled to 64 k points and apodized by Lorentzian line broadening of 0.30 Hz. The resulting spectra were manually phased and automatically baseline corrected using Topspin™ (Bruker Biospin) and the ppm scale was referenced towards the TSP peak at 0.00 ppm.

Since the composition and the concentration were very similar for every sample, the receiver gain was initially set at a fixed value in order to have a common intensity scale for all the acquired experiments. 

### 2.7. Pre-Processing of the NMR Spectra

Before conducting multivariate data analysis (chemometrics) the NMR spectra were cleaned and corrected from problems that commonly affect this type of data. First, the NMR regions between 15.21 and 9.20 ppm, between 6.34 and 4.59 ppm and between 0.62 and −5.61 ppm were removed because they included only noise or because the NMR signals of some metabolites (*i.e.*, urea, α and β anomeric sugars) were strongly affected by the residual HOD peak. Secondly, the spectra were corrected for misalignments in chemical shift primarily due to pH-dependent signals using the interval-based *icoshift* algorithm [15]. No binning was used for the whole chemometric approach, therefore full resolution spectra where used avoiding any loss of spectral information.

**Figure 2:**
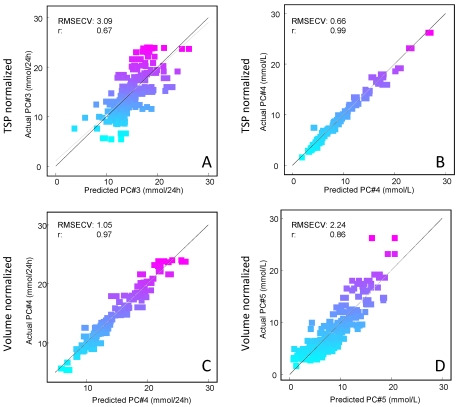
Four predicted *versus* measured plot of creatinine levels. (**A**) Perdeuterated 3-(trimethylsilyl) propionate sodium salt (TSP) normalized spectra predicting creatinine *excretion*; (**B**) TSP normalized spectra predicting creatinine *concentration*; (**C**) Volume normalized spectra predicting total creatinine *excretion*; (**D**) Volume normalized spectra predicting total creatinine *concentration*. The scores have been colored according to increasing creatinine concentration; where cyan corresponds to low concentration and magenta corresponds to high creatinine concentration.

In order to find the optimal normalizing method for pre-processing the urine NMR data, two different normalization procedures, TSP and volume normalization were tested to find the most appropriate PLS model. The PLS calibration models of creatinine are shown in Figure 2. 

The figure shows four actual *versus* predicted plots of PLS models predicting creatinine together with the model performances (prediction error and correlation coefficient). Figure 2A,B shows the result of PLS model of the total excretion of creatinine (mmol/24 h) (A) and the concentration of creatinine (mmol/L) (B) respectively, after normalizing the urine spectra according to the area of the TSP signals. Furthermore, the actual *versus* predicted plot of the total excretion of creatinine (Figure 2 and the concentration of creatinine (Figure 2D) are presented after volume normalization. The figure clearly demonstrates that TSP area normalization was more suitable when the aim was to predict the actual creatinine concentration (correlation coefficient, *r* = 0.99 and prediction error, RMSECV = 0.66 mmol/L). On the other hand the volume normalization was more suitable when the aim was to predict its total excretion (mmol/24 h), (correlation coefficient, *r* = 0.97 and prediction error, RMSECV = 1.05 mmol/L). In this study, the primary interest was the concentration of metabolites and therefore TSP normalization was chosen for correcting the NMR data. 

Out of 376 spectra, 112 samples were removed from the data set because of either outlying behaviour or inadequate PABA recovery. Twenty-two urine samples were considered as outliers due to presence of ethanol (1.17 ppm), paracetamol (acetaminophen) (2.18 ppm) and ibuprofen (0.91 ppm), which had been consumed by the subjects during the intervention period. Furthermore, the presence of acetate (1.91 ppm) indicated bacterial contamination [13]. The resulting 90 samples showed inadequate PABA recovery, indicating that the 24-h urine were not complete.

### 2.8. Statistics and Multivariate Data Analyses

Separate mixed linear models were used to assess the time-treatment interaction for the parameters BMI, protein, carbohydrate, fat, urinary nitrogen and urinary creatinine. Random effects were included to account for heterogeneity between subjects. The analyses were based on the 77 subjects (HP: *n* = 42 and LP: *n* = 35) who completed the 24 h urine collections at baseline, and at months 1, 3 and 6 during the dietary intervention period. It was tested whether gender, BMI, PABA, total urine volume, time, diet group or dietary components had a significant effect on the total urinary nitrogen or creatinine excretion. Where appropriate, variables that significantly affected urinary nitrogen or urinary creatinine were used as covariates in the MIXED analyses. Statistical analyses were performed with SAS (Statistical Analysis Package version 9.1 for Windows, SAS institute Inc., Cary, NC, USA) and the level of significance was *P* < 0.05. 

Multivariate data analysis in the form of principal component analysis (PCA) and partial least squares regression (PLS) was applied to the acquired and preprocessed NMR data to obtain optimal quantitative and qualitative information from the measured urine samples. PCA [16] is the fundamental approach in exploratory data analysis. With PCA, similarities and differences among samples can be highlighted and eventually groups of similarities and the variables (regions in the NMR spectra) involved in these differentiations can be inferred. This makes PCA a very useful unsupervised tool for a first exploratory approach to unknown data. The complex data matrix in PCA is reduced to few latent factors, *i.e.*, principal components (PC’s), sorted by significance (explained variance) which makes it easy to distinguish useful information from noise. 

Supervised exploration of dietary parameters was then performed using PLS, which is the primary tool for investigating quantitative information from NMR spectra. PLS is a multivariate calibration method by which two sets of data X (e.g., NMR spectra) and y (e.g., creatine) are related by means of regression. In order to group samples e.g., by their dietary intake, PLS discriminant analysis (PLS-DA) was chosen as a multivariate data analysis for classification. PLS-DA is a regression model describing maximum separation between two pre-defined classes and has been used to predict protein diet group [17]. 

Assignment of metabolites for an NMR spectrum of a biological sample is a highly complex task as resonances from all protons give rise to signals in the NMR spectrum. To investigate the influential areas of the spectra, the region selection method: interval PLS (iPLS) [18] proved to be very efficient. iPLS is an extension of PLS which develops local PLS models on a number of subintervals of the full spectrum region. The main advantage of iPLS is that it provides an overall picture of the relevant information in different spectral subdivisions, thereby removing interferences from other regions. iPLS reveals areas of the spectra which hold information about the reference data and is thus useful for interpretation and assignments. The summarizing iPLS plot shows the most informative region(s) of the NMR spectrum which had the lowest prediction error providing a straight comparison with the performance of the model obtained using the whole spectra area. Interval based chemometric models have previously been shown to be excellent exploratory tools in obtaining knowledge about informative regions of the NMR spectrum [13,19,20]. As for any other supervised classification method, careful validation is required in order to avoid overfitting the data. For the large data set this was achieved using a systematic cross-validation (CV) with five segments, leaving out one segment at a time from which the root mean square error of cross-validation (RMSECV) was calculated as a measure of the prediction error. However, for the small data sets, due to the low number of samples, full CV was used. 

For the intervals identified as potentially separating diet groups a confirmatory analysis of variance (ANOVA) based on the sums of maximum peak intensities was performed to statistically compare diet groups. The ANOVAs also included time (1, 3, 6 months) additively to adjust for possible time trends.

PCA was carried out on the dietary and effect variables at months 1 and 6 of the dietary intervention using Latentix 2.0 (www.latentix.com, Latent5, Copenhagen, Denmark). Also the NMR spectra were analyzed using the chemometric software LatentiX 2.0 while Matlab^®^ (2009, The Mathworks Inc., Natick, MA, USA) was used for the preprocessing (both normalization and signal alignment) of spectral data. The latter was performed using the interval-shifting, *icoshift* aligning tool [15]. iPLS and iPLS-DA were also performed in Matlab using the *iToolbox*. 

## 3. Results and Discussion

### 3.1. Dietary Intake

The demographic characteristics of the 77 subjects who completed the study and the mean dietary macronutrient composition during the 6 months diet intervention period are presented in Table 1. 

**Table 1:** Mean dietary macronutrient composition during the 6 months diet intervention period (both supermarket data and data provided from the 3-day dietary food diary *^1^*.

	HP diet	LP diet	*P ^2^*	*P ^2^*	*P ^2^*
	(*n* = 42)	(*n* = 35)	Main effect of time	Main effect of treatment	Time × treatment interaction
**Men/women**	21/21	12/23			
**Age** (year)	43.9 ± 4.9 *^3^*	42 ± 5.1			
**BMI **			<0.0001	NS *^5^*	NS
Baseline (kg/m^2^)	33.9 ± 1.1	34.6 ± 1.2			
Month 1 (kg/m^2^)	28.0 ± 1.1	28.0 ± 1.3			
Month 3 (kg/m^2^)	27.9 ± 1.1	29.6 ± 1.3			
Month 6 (kg/m^2^)	30.6 ± 1.2	30.9 ± 1.3			
**Energy intake **			<0.0001	NS	NS
Baseline (MJ)	10.1 ± 0.5	10.3 ± 0.6			
Month 1 (MJ)	6.8 ± 0.5	5.6 ± 0.6			
Month 6 (MJ)	5.6 ± 0.5	5.4 ± 0.6			
**Protein**			-	-	<0.0001
Aim	23–28	10–15			
Baseline (% E)	15.8 ± 0.6	17.5 ± 0.7			
Month 1 (% E)	24.1 ± 0.6	17.3 ± 0.4			
Month 6 (% E)	25.1 ± 0.7	18.1 ± 0.4			
**Supermarket**	26.6 ± 0.3	13.9 ± 0.3			
Months 0–6 (% E)				<0.0001	
**Carbohydrate**			-	-	<0.0001
Aim	45–50	57–62			
Baseline (% E)	46.9 ± 1.1	47.5 ± 1.3			
Month 1 (% E)	44.7 ± 1.1	56.3 ± 1.2			
Month 6 (% E)	46.9 ± 1.3	55.2 ± 1.4			
**Supermarket**	43.6 ± 0.3	56.2 ± 0.4			
Months 0–6 (% E)				<0.0001	
**Fat **			<0.0001	NS	NS
Aim	25–30	25–30			
Baseline (% E)	33.9 ± 1.0	32.8 ± 1.2			
Month 1 (% E)	28.9 ± 1.1	26.3 ± 1.2			
Month 6 (% E)	26.1 ± 1.2	25.3 ± 1.3			
**Supermarket**	25.8 ± 0.4	25.3 ± 0.4			
Months 0–6 (% E)				NS	
**U-Nitrogen ***^4^*			NS	0.001	NS
Baseline (g/24 h)	14.8 ± 0.6	14.8 ± 0.7			
Month 1 (g/24 h)	15.8 ± 0.6	11.4 ± 0.6			
Month 3 (g/24 h)	17.5 ± 0.6	12.8 ± 0.7			
Month 6 (g/24 h)	16.6 ± 0.6	13.8 ± 0.6			
**U-Creatinine ***^4^*			NS	0.078	NS
Baseline (mmol/24 h)	14.8 ± 0.5	14.4 ± 0.6			
Month 6 (mmol/24 h)	16.1 ± 0.7	13.9 ± 0.6			

*^1^* HP, high protein; LP, low protein. Supermarket data is based on the sum of energy provided from foods collected in the supermarket and from foods acquired from outside the supermarket. *^2^* Statistical analyses are carried out using PROC MIXED. *^3^ x* ± SE (all such values). *^4^* Adjusted for time, diet group, gender, protein intake, BMI, PABA and total urine volume. *^5^* NS, non significant.

No differences in baseline data were found and the BMI of the subjects did not differ significantly between the two groups (HP and LP). There was a main effect of time indicating that both diet groups had a significant weight loss during the 8-week LCD but no significant weight change between groups occurred during the 6-month intervention. The actual dietary intake complied with the stipulated diet in the two groups, both as reported in the food diary and according to the data obtained from the supermarket recordings. Validation of the self-reported dietary intake was performed by assessment of the total 24-h urinary excretion of nitrogen and creatinine. The total excretion of nitrogen (at months 1, 3 and 6, *P* < 0.0001) and a tendency towards the total excretion of creatinine (month 6, *P* < 0.078) were significantly higher for the HP diets compared to the LP diets. 

**Figure 3:**
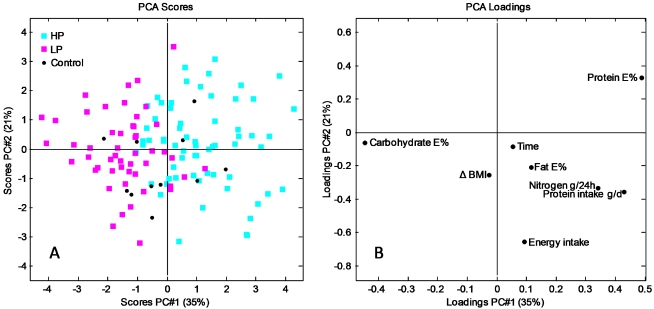
(**A**) Principal component analysis (PCA) scores plot for the subjects at month 6 colored according to diet protein level; (**B**) Corresponding PCA loading plot of the dietary variables (total energy intake (MJ/day), carbohydrate (% of energy), protein (% of energy), protein intake (g/day), fat (% of energy), and the effect of variable nitrogen (g/24 h) at month 6. ΔBMI is calculated as the BMI change between month 6 and month 1. The loading plot illustrates the contribution of each variable to each PC.

In order to provide an overview of the relationship between diet and effect variables at the subject level during the 6 months intervention, a PCA model was calculated using the dietary data and the corresponding scores and loading plot are shown in Figure 3. 

The first two principal components (PC’s) describe 56% of the variation. The score plot reveals grouping of the subjects according to diet protein group where the subjects following the LP diet are located in the negative region of PC1 and the subjects following the HP diet are mainly present in the positive region. The subjects on the control diet are situated between the two diet groups as expected. Inspecting the first PC using its loading plot it emerges that PC1 describes variation in dietary energy with protein intake (total intake (g/day) and % of energy) in the positive direction of PC1 and carbohydrate (% of energy) in negative direction and these variables are responsible for the division of the diet groups. PC2 is explained by variation in energy intake in the negative direction of PC2 and protein (% of energy) in the positive direction. Time and fat (% of energy) are correlated but do not have a remarkable influence on the model. Total nitrogen excretion (mol/24 h) is closely correlated to protein intake (g/day). 

### 3.2. Validation of NMR Data by Means of Creatinine and Nitrogen Calibration

**Figure 4:**
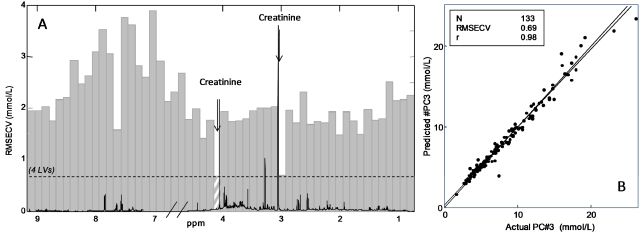
(**A**) Interval PLS (iPLS) plot of the prediction of creatinine concentration (mmol/L) obtained on ^1^H NMR spectra of urine using 54 intervals (200 variables). The dotted line shows root mean square error of cross validation (RMSECV) (4 latent variables (LV’s)) for global model. The intervals containing signal from creatinine (4.02–4.15; 2.95–3.05 ppm) show the lowest prediction error; (**B**) Predicted *versus* measured plot of PLS model calculated on the hatched intervals using 3 LV’s.

In order to investigate and enhance the predictive models of urinary creatinine and nitrogen, iPLS models were developed. The iPLS model for prediction of urinary creatinine concentration is shown in Figure 4A. The iPLS model was calculated with 54 intervals, each made of 200 variables, and the root mean square error of cross validation (RMSECV) of each model is shown for each interval. The iPLS plot shows the chemical shift axis in ppm *versus* RMSECV together with the average spectrum. The dotted line represents RMSECV using 4 latent variables (LVs) for the global model identical to the model in Figure 4B. The interval containing a large signal at 4.06 ppm has the lowest prediction error compared to the global model and the interval with the second lowest RMSECV is the interval containing the large singlet at 3.05 ppm. Both these intervals include signals from creatinine. The best iPLS model, using 3 LV’s components around 4.06 ppm, is illustrated in Figure 4B, where the predicted *versus* reference values (mmol/L) are plotted. The correlation coefficient of the model was high (*r* = 0.98) and with a low prediction error (RMSECV = 0.69 mmol/L). 

**Figure 5:**
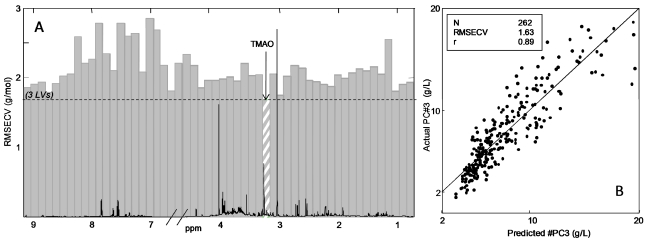
(**A**) iPLS plot of the prediction of nitrogen concentration (mmol/L) obtained on ^1^H NMR spectra of urine using 54 intervals (200 variables). The dotted line shows RMSECV (3 latent variables (LV’s)) for global model. The interval (3.17–3.29 ppm) containing signal from trimethylamine-*N*-oxide (TMAO) shows the lowest prediction error; (**B**) Predicted *versus* measured plot of PLS model calculated on the hatched intervals using 3 LV’s.

Correspondingly an iPLS model on the prediction of urinary nitrogen concentration was calculated, and the result is shown in Figure 5A. The model is calculated with 54 intervals of 200 variables each and in this case the global model uses 3 LVs as the optimal number of components. The iPLS plot reveals that the interval at 3.27 ppm gives the lowest prediction error, which is the interval that includes a signal from trimethylamine-*N*-oxide (TMAO) (hatched interval Figure 5A). The predicted *versus* measured plot from this interval is illustrated in Figure 5B. The correlation coefficient of the model was high (*r* = 0.89) and with a low prediction error (RMSECV of 1.63 g/L). 

One finding of the present study revealed that the TMAO signal in the NMR spectra was highly correlated to daily urinary nitrogen excretion. Urinary nitrogen is a well established biomarker of total daily protein consumption [14,21,22] and it is thus interesting that urinary TMAO can act as a marker associated with the quantity of nitrogen and thereby consumed protein. Recent metabolomic research has identified a variety of biomarkers of diets rich in protein and our findings seem to corroborate previous studies [8,23,24,25]. Especially the excretion of TMAO has in several studies shown to be elevated after consumption of fish and high-meat diets [8,23,26]. Fish contains high levels of TMAO which is naturally synthesized as an antifreeze agent in many types of deep sea fish and thus accumulates in the tissues [27]. In a study by Lenz *et al*. [23] metabolomic urinary profiles collected in British and Swedish populations were compared. The urinary profiles were clearly prone to variability due to dietary and lifestyle influences. An increased urinary excretion in TMAO was observed in the Swedish population as a consequence of a fish-diet, which was not found in the British population. Dumas *et al*. [26] conducted a large-scale epidemiological metabolomics study on biochemical variability of urinary profiles from populations in Japan, North America, and China. They found that high urinary concentrations of TMAO were particularly dominant in the Japanese population, consistent with their high dietary intake of fish. However; to our knowledge, the present study is the first to highlight the quantitative relationship between reference measurements of creatinine and nitrogen and the NMR metabolomic profile. This study showed that TMAO concentration was associated with urinary nitrogen excretion and thereby with protein consumed. Since TMAO is present in a high concentration in fish and meat it is assumed that this correlation only is valid for animal protein sources which may be important in the search for biomarkers of specific protein intake.

### 3.3. ^1^H NMR Spectroscopy of Human Urine

**Figure 6:**
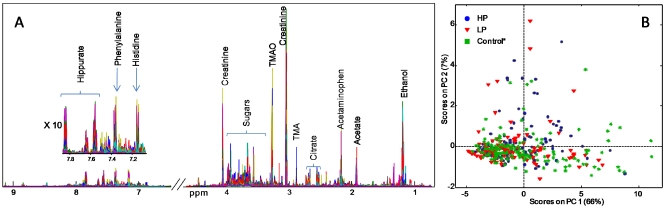
(**A**) ^1^H NMR plot of the complete urine spectra (*n* = 264) from 0.62–9.20 ppm including assignments of the most dominant signals; (**B**) PCA model of ^1^H NMR spectra showing the overall variation between the samples. The PCA scores are colored according to diet group (high protein (HP), low protein (LP) and controls) showing no grouping at the individual level according to diet.

A ^1^H NMR line plot of the complete urine spectra (*n* = 264) is shown in Figure 6A together with assignments of the major metabolites [28,29,30,31]. The figure shows the great complexity of ^1^H NMR spectra of urine as many signals show large variations between the samples. In order to investigate the spectra about the diet groups a PCA model of the entire ^1^H NMR dataset was calculated and the overall variation is illustrated in the score plot shown in Figure 6B. 

The samples in the PCA score plot are colored according to diet group (HP or LP collected at months 1, 3 and 6, respectively) and include the baseline samples and control samples. This plot reveals large intra- and inter-individual variations and no groupings at the individual level can be observed according to diet intervention. 

**Figure 7:**
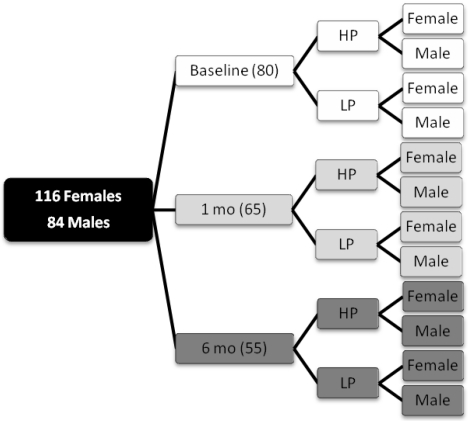
Averaging design; 104 spectra are reduced to 12 averaged spectra, without controls and only including subjects with all four visits and a PABA recovery of min 75%. The 12 averaged spectra are divided into diet group (HP or LP) and gender at baseline, months 1 and 6.

In order to remove the large inter-individual variation between the samples as shown in Figure 6B, the ^1^H NMR spectra were pooled to obtain averages of diet groups and remove large unwanted variation. The urine spectra were pooled according to the design shown in Figure 7. Only subjects (*n* = 26) from whom samples have been taken at all 4 time points are included in the averaging. Using this design the 104 individual spectra were reduced to 12 averaged divided into diet group and gender at baseline, months 1 and 6 of the dietary intervention period. 

### 3.4. Associations Between Dietary Patterns Related to Protein Intake and Human Urinary Metabolites

**Figure 8:**
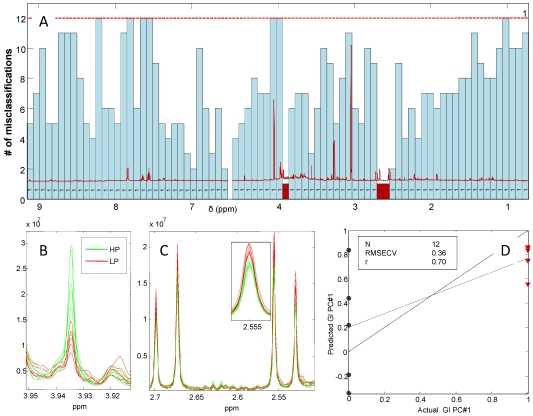
(**A**) iPLS-DA plot of classification of the high protein (HP) and low protein (LP) diet from 12 averaged ^1^H NMR spectra of urine calculated on 80 intervals (full CV) showing the number of misclassification in absolute numbers. The 12 averaged spectra are divided into diet group (HP or LP) and gender at months 1, 3 and 6. The red dotted line indicates the number of misclassification for the full spectral model together with the number of PC. The RMSECV of the global model calculated with 1 LV’s is markedly improved by the intervals colored red (3.86–3.94 + 2.45–2.72 ppm). These intervals include signals from creatine (3.93 ppm); (**B**) The RMSECV of the global model calculated with 1 LV’s is markedly improved by the intervals colored red (3.86–3.94 + 2.45–2.72 ppm). These intervals include signals from creatin (3.93 ppm); (**C**) These intervals include doublets from citric acid (centered at 2.86 and 2.54 ppm); (**D**) The actual *versus* predicted plot shows almost perfect separation of the two diet groups using the selected intervals.

To investigate the effect of dietary patterns on the urinary metabolic profiles at group level, an iPLS-DA model of 12 averaged spectra from months 1, 3 and 6 was developed. The model was calculated on 80 intervals of 135 variables each allowing the use of only 1 LV and yielding 12 misclassifications for the global model (Figure 8).

Figure 8 shows two interesting intervals which are able to improve the number of misclassification of the global model markedly. These intervals which are colored red (3.86–3.94 + 2.45–2.72 ppm) include signals from creatine (3.93 ppm) and the doublets from citric acid (centered at 2.68 and 2.54 ppm). Inspection of these metabolite signals shows that the discrimination of the HP and LP groups is due to the significantly higher creatine intensity of the HP group (*P* = 0.002) together with a significantly lower citric acid content of the urine (*P* = 0.03) and the reverse behavior was seen for the LP group. Furthermore, a tendency towards an increased urinary TMAO with the HP diet was observed (figure not shown). The model performance is shown in Figure 8D and reveals an almost perfect separation of the two averaged groups. The predicted *versus* measured plot shows that apparently the HP group (actual value = 0) has a higher deviation compared to the LP group (actual value = 1) which are predicted more precisely.

In the current study the variability of the metabolic phenotypes of urine samples from subjects of both genders following controlled long-term HP or LP diets was investigated. Urinary creatine was increased in subjects on HP diets but also a tendency of an increased urinary TMAO was found which is in agreement with several other studies investigating biomarkers of HP diets [8,23]. It was suggested that the alkali load provided by a high intake of fruit and vegetable increases the intracellular pH, meaning that the tubular re-absorption of citric acid decreases and its excretion increases [32]. 

### 3.5. Associations Between Dietary Patterns Related to Protein Intake and Gender-Specific Metabolites

The 12 averaged spectra from months 1, 3 and 6 were further divided into male and female urine spectra and colored according to diet group. The discriminative signals, as observed by visual inspection, were assigned according to the literature [19,28,31,33,34]. A table of these signals are listed in Table 2. The table reveals several complementary metabolites associating with the HP or LP diets on the group level.

**Table 2:** Identified gender-specific urinary metabolites related to protein intake.

δ ppm	Female	Male	Chemical assignment ^a^
9.13		HP	Trigonelline (from Caffeine)
8.84 (triplet)		HP	Trigonelline (from Caffeine)
8.46		HP	Formate
4.34	LP		Tartaric acid (grape/wine)
3.93	HP	HP	Creatine
3.88 (doublet)	LP		Mannitol
3.84 (doublet)	LP		Mannitol
3.75-3.79	LP		Alanine
3.57	LP		Glycine
3.43 (triplet)	HP	HP	Taurine
3.27	HP		TMAO (trimethylamine-*N*-oxide)
3.21		HP	Carnitine
3.04	HP	HP	Creatine
2.92	LP		(dimethyl-glycine)
2.88	HP		TMA (trimethylamine)
2.73	LP		DMA (dimethylamine)
2.68 (doublet)	LP	LP	Citric acid
2.54 (doublet)	LP	LP	Citric acid
2.41		LP	Succinate
2.32		HP	Taurine
2.20		HP	Carnitine
2.27 (triplet)	HP		Taurine
2.22		LP	Ribose
2.06	LP		*N*-acethyl glycosamine (NAG)
1.48 (doublet)	LP		Alanine

^a^ Several other NMR signals proved important for gender discrimination but have not been reported because not chemically assigned.

The differentiation between the HP or LP males and HP or LP females revealed alternative metabolites describing the difference between protein sources. HP-female urine was characterized by TMAO, TMA, and a number of unidentified matabolites, whereas HP-males had a greater urinary taurine, carnitine, formate and trigonelline (from caffeine). LP-females had an increased excretion of tartaric acid (from grape/wine), mannitol, alanine, glycine and NAG, whereas LP-males had an increased excretion of succinate and several unidentified metabolites. Xu *et al.* found that the most influential low molecular weight metabolites characterizing the vegetarians (relative to the omnivorous diet group) were increased NAG, succinate, citrate, hippurate and glycine and decreased TMAO, taurine, formate, phenylalanine and methylhistidine [9]. Most of these findings except from phenylalanine and methylhistidine were confirmed in this study. However, it remains elusive how these differences arise from different protein sources consumed in males and females primarily because of the lack of sufficient robustness of the markers. What is more, the gender distribution showed to be rather unbalanced. In the high protein group the gender distribution seemed appropriate, but in the low protein group two times more women were found which should be taken into consideration.

## 4. Conclusions

In the present study a holistic metabolomics approach was applied with no *a priori* selection of metabolites. Thereby it was possible to make an explorative investigation of the influence of dietary protein exposure on the metabolite profiles at the group level. The study was conducted under highly controlled conditions including repeated 24 h urine collections after 1, 3 and 6 months of the dietary intervention period and 3-day dietary recordings were collected at the corresponding 3 time-points combined with a controlled dietary assessment during the entire 6-month period using the supermarket model. The energy intake during the 6 months dietary intervention was *ad libitum* in order to investigate the impact of the diets on weight maintenance after the LCD. 

The study revealed a highly consistent individual correlation between the metabolic profiles and the total urinary nitrogen (*r* = 0.89) and urinary creatinine (*r* = 0.98). This is considered a major result since urinary nitrogen is a well-established biomarker for daily protein consumption and since such a high correlation firmly establishes the validity of the exploratory metabolomics approach. Not only can the NMR metabolic profiles provide the total nitrogen and the creatinine content, it can also be used for validation of and fine tuning of the NMR metabolomics approach and finally give additional and simultaneous information about other metabolites and biomarkers that may be relevant for the given application. 

The major limitation of the *ad libitum* free-living 6-month dietary intervention design used in the present metabolomics study was the lack of consistency in the diet consumed during the 3-day dietary recordings, both within each diet group, within each participant at the 3 different time-points, and within the 3 days of dietary recording. As a matter of fact, the 3-day dietary recordings showed a decreased energy intake during the intervention most probably caused by energy under-reporting which is a common issue in self-reported dietary assessment methods [3,4,35,36] or simply because the subjects have been used to the restricted calorie intake during the LCD. As a result of the *ad libitum* intervention design, prediction of protein intake or identification of metabolites relating to single individual food items using the data from the 3-day dietary recordings was not successful. Nevertheless, differentiated metabolite profiles at the group-level in the HP and LP diet groups was observed due to the quantity of protein eaten as a result of the changes induced by long-term exposure. Thus, these markers can be interpreted as more general markers, not being confounded by markers from single dietary factors.

Based on the results of the present study, it is recommended that additional validation of the potential of TMAO is generated in order to assess its suitability for ranking individuals by their level of protein intake. Furthermore, application of complementary analytical techniques with higher sensitivity such as LC- or GC-MS will be necessary to identify more detailed metabolite patterns and possibly to reach a more individualized level reflecting the dietary patterns imposed here. 
